# Effects of Pravastatin in Adriamycin-Induced Nephropathy in Rats

**Published:** 2018

**Authors:** Esrafil Mansouri, Mohammad-Ali Assarehzadegan, Fereshteh Nejad-Dehbashi, Wesam Kooti

**Affiliations:** a *Cellular and Molecular Research Center, Department of Anatomical Sciences, Faculty of Medicine, Ahvaz Jundishapur University of Medical Sciences, Ahvaz, Iran. *; b *Department of Immunology, Faculty of Medicine, Iran University of Medical Sciences, Tehran, Iran.*; c *Cellular and Molecular Research Center, Ahvaz Jundishapur University of Medical Sciences, Ahvaz, Iran.*; d *Cellular and Molecular Research Center, Sabzevar University of Medical Sciences, Sabzevar, Iran.*

**Keywords:** Nephropathy, Adriamycin, Pravastatin, Nephroprotective, Rat

## Abstract

The aim of this study is to evaluate the effects of pravastatin on Adriamycin (ADR)-induced nephropathy and the mechanisms involved. Forty rats were divided into the following 4 groups: control, ADR (15 mg.kg^-1^, IP), ADR plus pravastatin (20 mg.kg^-1 ^which was started 5 days prior to ADR injection), and ADR plus pravastatin (20 mg.kg^-1 ^which was started 5 days after ADR injection). On day 20 after ADR injection, the animals were sacrificed. The results showed that administration of pravastatin decreased the levels of 24-h urinary protein (24-h UP), blood urea nitrogen (BUN), and creatinine (*p* < 0.05) which had increased after the injection of ADR; in addition, pravastatin reversed structural changes seen in ADR group. Furthermore, pravastatin elevated mRNA and protein expression of nephrin (*p* < 0.05) which had been reduced in ADR group. We conclude that pravastatin protects and treats renal injury induced by ADR.

## Introduction

Adriamycin (ADR) or Doxorubicin is a wide spectrum anticancer anthracycline antibiotic that is widely used in treating cancers such as hematological malignancies and solid tumors. However, the application of ADR has been limited due to the incidence of dose-dependent toxicities in some vital organs such as liver, heart, and kidneys. The exact mechanisms of renal injury induced by ADR are not yet fully known (1-3). In experimental studies, ADR-induced nephropathy is accompanied by hypoalbuminemia, hypercoagulability, dyslipidemia, proteinuria, edema, and ascites formation (4). The common feature in this type of nephropathies (experimental nephropathy) is a toxin which induces a non-inflammatory podocyte foot process disunion that creates focal segmental glomerulosclerosis (FSGS) and tubulointerstitial damage, leading to nephropathy (5). It is well known that podocytes have a vital role in the formation of slit diaphragms (SD). Therefore, it is clear that podocyte damage causes proteinuria. Nephrin, one of the most important podocyte-associated proteins, helps maintain the integrity of SD and prevents the development of proteinuria. Hence, this proves that nephrin plays the main role in keeping the constructional and functional completeness of the glomerular filtration barrier and the development of proteinuria (6). Defects in nephrin expression lead to the development of different forms of proteinuria such as inherited and acquired proteinuria (7). HMG-CoA reductase inhibitors (statins) are lipid-lowering factors widely used in the treatment of high low-density lipoprotein (LDL) levels. It is generally accepted that mechanisms beyond the reduction of cholesterol contribute significantly to the antiatherogenic and tissue-protective properties of statins (8). Recently *in-vivo *investigations have suggested that statins also have reno-protective properties independent from their lowering cholesterol effects. For example, treatments of acute renal failure induced by ischaemia-reperfusion by statins were impressive, statins were effective as well in treating tubulointerstitial nephritis induced by administration of chronic cyclosporine or unilateral ureteral obstruction (9). The useful effects of statins on glomerulopathy have also been indicated in several studies. It is proved that employing statins will also be effective in the treatments of anti-Thy1 glomerulonephritis, Heymann nephritis, streptozotocin-induced nephropathy, and nephrotoxic serum-induced nephritis which are murine models of mesangial proliferative nephritis, membranous nephropathy, diabetic nephropathy, and crescentic glomerulonephritis, respectively (10, 11). Above all, many studies have reported that statins ameliorate glomerulopathies through their protective effects on podocyte (12). Collectively, these data suggest that statins may be effective in glomerulopathies triggered by podocyte injury. In this experiment, we have investigated the effects of pravastatin on an animal model of nephropathy induced by adriamycin.

## Experimental

*Animals and experimental design*

In this experimental study 40 male Sprague Dawley rats, weighing 150 to 170 g, were utilized. They were procured from the central animal house of Ahvaz Jundishapur University of Medical Sciences, Ahvaz, Iran. The protocol used in these studies was approved by the Ethics Committee of Ahvaz University of Medical Sciences. The animals were maintained under controlled conditions of temperature 21 ± 2 °C and a 12/12 h light/dark cycle and were allowed free access to standard rat chow and tap water ad libitum. They were divided into four groups of 10 animals each:

Group 1: Control; received distilled water orally

Group 2: ADR; Intraperitoneal injection (IP) with ADR (15 mg.kg^-1^; Sigma Chemical Co. St. Louis, USA)

Group 3: Pravastatin plus ADR; treatment with pravastatin orally (20 mg.kg^-1 ^dissolved in distilled water; Sigma Chemical Co. St. Louis, USA) which was started 5 days prior to ADR injection ADR and continued until the end of the experiment (12). 

Group 4: ADR plus pravastatin; treatment with pravastatin orally (20 mg.kg^-1^ dissolved in distilled water); it was started from the beginning of the fifth day after ADR injection, since we found a significant increase in urinary protein, and it continued until the end of the experiment (1). On day 20 after ADR injection, urine was collected for 24 h, using a metabolic cage to determine the 24-h urinary protein (24-h UP) by commercially available kits (Pars Azmon, Iran). On the following day, six rats were selected randomly from every group and were then sacrificed under ketamin anesthesia, and their right kidneys were rapidly removed for histopathological and the left kidneys were cut into 2 parts for molecular studies.

*Renal function*

After anesthesia, blood samples were collected from the left ventricle of heart, centrifuged at 4000 rpm for 20 min. To determine of renal function, blood urea nitrogen (BUN) and serum creatinine were measurement by auto analyzer (Vitalab Selectra E, Netherland) based on the manufacturer’s protocol of colorimetric diagnostic kits. 

*Histopathology*

 The kidneys were fixed in formalin (10%), dehydrated through increasing concentrations of ethanol, and were embedded in paraffin. The sections (5 µm) of kidney tissues were stained with hematoxylin and eosin and were assessed by light microscope. Histopathlogical evaluations were accomplished by one of the researchers.

*Expression of nephrin mRNAs in the renal cortex*

In this study, the amount of the nephrin mRNAs expression in the renal cortex, was determined by real-time quantitative fluorescence PCR with SYBR Green and also, glyceraldehyde-3 phosphate dehydrogenase (GAPDH) was utilized as an internal control for standardization of mRNA level. Table 1 showed primers that we used (13). Briefly, the kidneys were cut along the sagittal plane. Then, cortex tissue was isolated from the medulla of each kidney under magnification with a dissecting microscope and total RNA was extracted from the cortex using RNeasy Plus Mini Kit (Qiagen, USA) based on the manufacturer’s protocol. Then, 1 μg of total RNA prepared were utilized to synthesize cDNA with AccuPower^®^ CycleScript RT PreMix (Jena Bioscience GmbH, Germany) according to the manufacturer’s protocol. The reaction conditions were 42 °C for 1 h and then at 95 °C for 5 min. Then, 5 μL of cDNA were added to the 45 μL reaction mixture (contain master mix and primers 10 µM), after that, real-time PCR was performed using real-time PCR detection system (Roche- Light Cycler 96, Germany). The PCR conditions were: predenaturation at 93 °C for 3 min, 40 cycles of denaturation at 93 °C for 30 sec, annealing at 55 °C for 45 sec, and extension at 72 °C for 45 sec. Expression of nephrin gene was normalized to that of GAPDH. mRNA was calculated by subtracting the reference gene from target gene: 

ΔCT = CT_target gene_ - CT_reference gene_

ΔΔCT = ΔCT_test group _- ΔCT_control group_

**Table 1:** Primer sequences for real-time PCR

**Gene**		** Sequences bp**		
Nephrin (NM_022628.1)	Forward Reverse	-CCCTCCGGGACCCTACTG -TCTGGGAGGATGGGATTGG	82
GAPDH (NM_017008.4)	Forward Reverse	-TGGTCTACATGTTCCAGTATGACT -CCATTTGATGTTGGCGGGATCTC	134

*Western blotting*

Western blotting was carried out as explained previously (14). Briefly, the kidneys cortex tissue were homogenized in lysis buffer [50 mM Tris_Cl (pH 7.5), 150 mM NaCl, 1% Triton X-100, 0.25% wt/vol sodium deoxycholate, 0.1% SDS, 1 mM EDTA, 1% protease inhibitor cocktail (Roche, Mannheim, Germany)]. Homogenates were centrifuged at 3,000 rpm for 15 min at 4 °C, and the protein concentration of the lysate were determined using the Bradford method (bovine serum albumin as standard) (15). Seventy-five μg of total proteins were separated on 8% SDS-PAGE transferred to PVDF membrane. The membrane was incubated with blocking buffer (5% skimmed milk in TBS-T) and incubated by primary antibody (1:500 dilution) of nephrin (goat polyclonal IgG) (Santa Cruz Biotechnology, USA) overnight at 4 °C. Then, membrane incubated with hersradish peroxidase conjugated secondary antibody (donkey anti-goat IgG 1:5000 dilution) (Santa Cruz Biotechnology, USA). Finally, protein was detected by ECL kit (Najm Biotech ECL, Iran) and was visualized using ChemiDoc^™^ XRT^+^ system (BIO-Rad Lab).

*Statistical analysis*

All results were expressed as the mean ± SD. Statistical significance was assessed with one-way ANOVA by SPSS version 15 (IBM, USA) for Windows followed by Tukey’s post-hoc tests. *p* < 0.05 was assumed as statistically significant.

## Results

*Effect of pravastatin on 24-h UP, BUN and Creatinine*

As shown in Table 2, Rats receiving ADR showed significant increase in 24-h UP, BUN, and creatinine levels when compared to control group (*p* < 0.05) but, administration of pravastatin in groups of 3 and 4 decreased levels of 24-h UP, BUN, and creatinine (*p* < 0.05). Also, we didn’t observe any difference between groups 3 and 4 (*p* > 0.05).

**Table 2:** Effect of pravastatin on 24 h UP, BUN and serum creatinine in ADR- induced nephropathy in rats

**Groups **	**24-h UP (mg/24 h) **	**BUN (mg/dL) **	**Creatinine (mg/dL) **
Control (1)	5.76 ± 1.55	8.15 ± 1.49	.67 ± .1
ADR (2)	39.12 ± 3.15*	50.98 ± 4.72*	1.94 ± .18*
Pravastatin + ADR (3)	17.61 ± 2.12**	15.11 ± 2.84**	.85 ± .12**
ADR + Pravastatin (4)	14.86 ± 2.36**	18.88 ± 2.67**	.71 ± .12**

Values were expressed as mean ± SD, n = 6.

* *p < *0.001, *vs*. control.

** *p < *0.001 *vs. *ADR group.

*Effect of pravastatin on renal histopathology*

**Figure 1:**
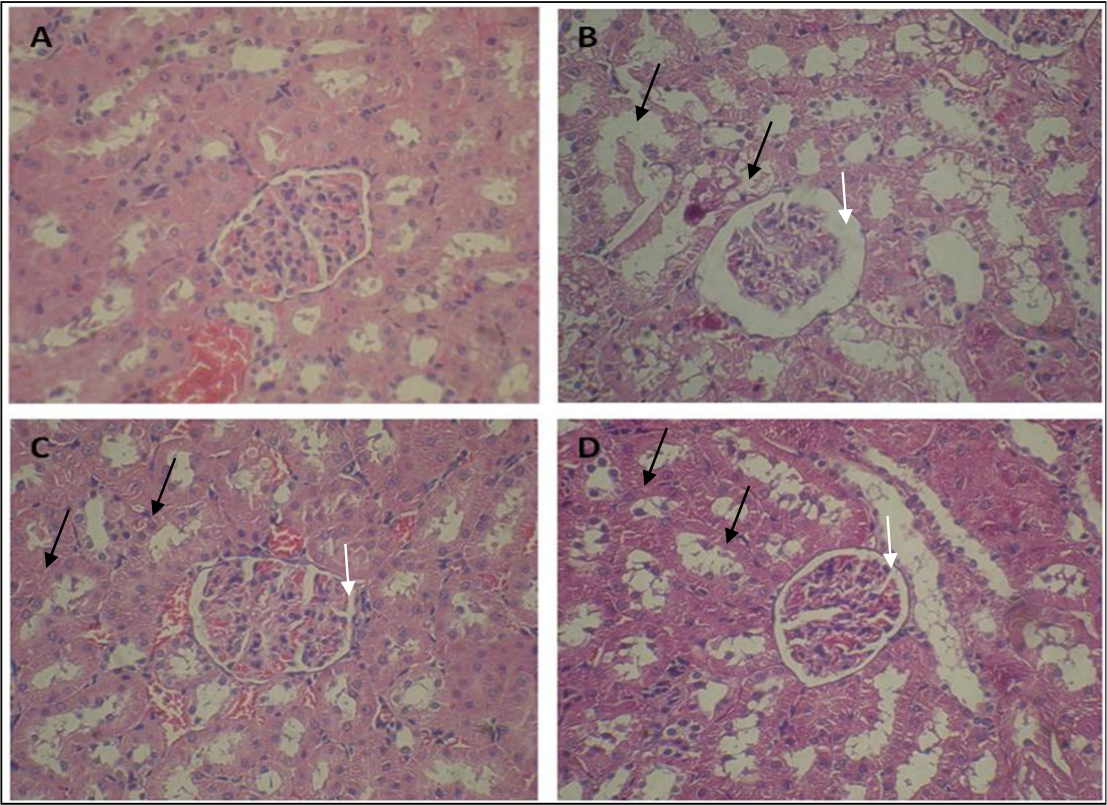
Effect of pravastatin on ADR-induced nephropathy rats. Microscopic slide of rat kidney (H and E *× *300). (A) Control group with normal structure of glomeruli and cortical tubules and (B) ADR group with degeneration of the epithelial cells of some tubules (black arrow) and dilated bowman’s space (white arrow). (C and D) Pravastatin administration reduced these changes

Histopathological evaluation showed that control group had normal structure in glomeruli and tubules (Figure 1A). Whereas, ADR-treated group indicated dilated urinary space in renal corpuscle and destruction of renal tubules, desquamated epithelial cells of tubules, and cystic formed in the cytoplasm of tubule cells (Figure 1B). Administration of pravastatin in 3 and 4 groups revealed restoration of normal structure of renal corpuscle, regeneration of tubules and renal epithelial tubules (Figures 1C and 1D). However, we didn’t find any differences between the groups of 3 and 4 in structure of kidney. 

*Effect of pravastatin on mRNA and protein expression of nephrin*

**Figure 2:**
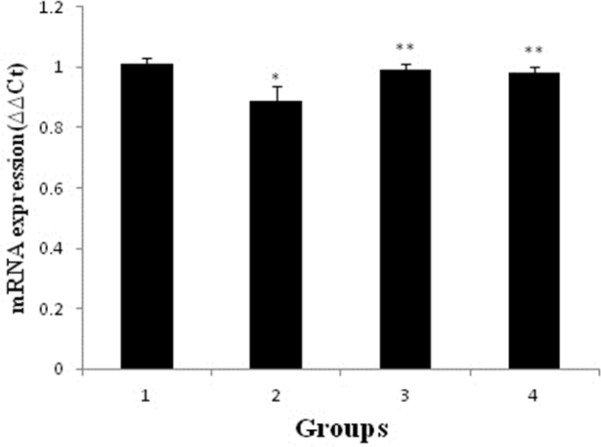
Effects of pravastatin on mRNA expression of nephrin in different groups (six rats for each group). 1 (control); 2 (ADR); 3 (pravastatin+ ADR) and 4 (ADR+ pravastatin). ^*^p < 0.05, *vs.* control. ^**^p < 0.001, *vs. *ADR group

**Figure 3:**
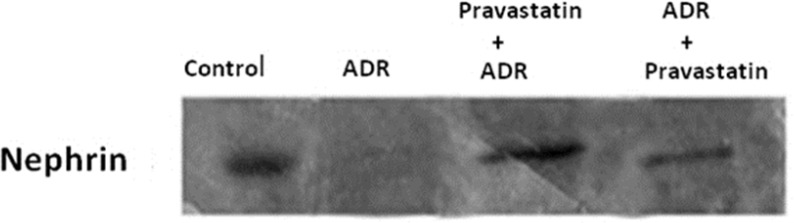
Effects of pravastatin on nephrin expression in different groups (six rats for each group). Western blotting demonstrated the expression of nephrin in control group. The expression of this protein decreased in ADR group, but pravastatin partially restored nephrin expression

As shown in Figures 2 and 3, mRNA and protein expression of nephrin in group receiving of ADR significantly reduced when compared to control group (*p* < 0.05). But, pravastatin in groups of 3 and 4 could increase mRNA and protein expression of nephrin (*p* < 0.05) and there was no difference between 3 and 4 groups (*p* > 0.05).

## Discussion

Our study demonstrated that pravastatin reduced urinary protein excretion and retained the renal function and expressions of nephrin in ADR-induced nephropathy rats. Additionally, the structure changes of renal tissue were partially recovered by pravastatin. We found that pravastatin significantly reduced proteinuria with ADR-induced renal injury as well. Proteinuria is a major marker of damaged glomerular filtration barrier. Podocyte is the last barrier in glomerular filtration membrane (16, 17). After irritation with various agents, the podocytes may be separated and excreted with urine. The injury of podocytes is one of main factors in the incidence of proteinuria (18). Documents indicate that statins reduce proteinuria and improve renal function (19, 20). It is probably for this reason that statins maintain podocyte integrity and consequently the pathogenic effects of a tubular load of ultrafiltered proteins (21), which confirms the results of the present study. In the present study, BUN and creatinine levels indicated a significant increase in ADR group, which confirms the occurrence of renal dysfunction. It was demonstrated that the oxidant damage induced by ADR with generation of reactive-oxygen-species (ROS) raises urea and creatinine levels in serum (22). Similar to the previous study (23), our study showed that pravastatin significantly diminished ADR-induced increase in BUN and Cr. We also found that pravastatin improved structural changes that were induced by ADR. HMG-CoA reductase inhibitors such as pravastatin have been suggested to improve renal damage in many experimental models of nephropathy (21, 24-26). This feature of statins may be due to an inhibitory effect on the generation of free radicals. Furthermore, it is suggested that statins have been shown to have beneficial effects on monocyte recruitment, mesangial cell proliferation, endothelial function, renal hemodynamics, and mesangial matrix accumulation as well as anti-inflammatory and immunomodulatory activities. In theory, each of these mechanisms might mediate the assumed renoprotective properties of statins (26-28). In our experiment, diminished glomerular nephrin expression improved via pravastatin. Nephrin, which is a main component of the filtration barrier, is expressed on the filtration slit diaphragm in the renal glomerulus. Downregulation of nephrin expression in glomeruli demonstrates a defect in the glomerulus to have proper filtration. As a result, reduction of abnormal nephrin expression in renal glomerulus induces proteinuria and the development of nephropathy, and the improvment of nephrin expression reduces proteinuria (18, 29 and 30). Several previous studies indicated that the reduced expressions of nephrin in renal injury were restored by statins (12, 31), which is consistent with our results. 

## Conclusion

This study demonstrated that pravastatin can protect and treat structural and functional damages

of kidney against ADR. Our data provided a potential rationale for the clinical application of pravastatin to prevent and reduce of ADR-induced renal injury.
